# How do wound care nurses structure the subjective frame on palliative wound care? A Q-methodology approach

**DOI:** 10.1186/s12912-022-00900-7

**Published:** 2022-05-17

**Authors:** Ye-Na Lee, Sung Ok Chang

**Affiliations:** 1 Department of Nursing, The University of Suwon, Hwaseong, Republic of Korea; 2 College of Nursing and BK21 FOUR R&E Center for Learning Health Systems, Korea University, 145, Anam-ro, Seongbuk-Gu, Seoul, 02841 Republic of Korea

**Keywords:** Palliative wound care, Palliative care, Wound care, Q-methodology, Subjectivity

## Abstract

**Background:**

Palliative wound care is important for stability in terminal care. It addresses both the physical and psychological needs of patients and facilitates other aspects of terminal care. Appropriate competencies of nurses regarding palliative wound care can improve patient outcomes and raise their quality of life. The purpose of this study was to identify how wound care nurses structure the subjective frames regarding palliative wound care.

**Method:**

This study utilized Q-methodology to analyze their subjective viewpoints. Forty nurses experienced in palliative wound care were asked to completely classify 35 Q-statements into a normal distribution shape. The PQ-Method program was used to conduct principal factor analysis and varimax rotation for data analysis.

**Results:**

This study revealed 4 Q-factors of palliative wound care: “Focusing on care within the boundary of current patient demands,” “Comparing continuously the priorities on wound healing and disease care,” “Preparing and preventing from worsening via tracking care in advance,” and “Moving forward with a clear direction by confronting the declining condition.”

**Conclusion:**

We hope that the results of this study are used in the development of nursing education that reflects professional perspectives of palliative wound care, thus helping to improve nursing competencies in palliative care.

## Background

Advancements in medical technology have extended the average lifespan through significant contributions to overall health levels and disease recovery. With such technology allowing for longer lives, more people spend their last moments in medical facility [1]. Furthermore, the importance of end-of-life care has risen due to a global focus on the social atmosphere and policies that protect patients’ right to self-determination, as well as their dignity and value as human beings [2]. End-of-life care is a holistic approach to care that involves caring for end-of-life patients and their families, maintaining their dignity and high quality of life as human beings, and fulfilling their physical, psychological, and spiritual needs to ensure that the patients are comfortable through the last moments of their lives [3].

The skincare of patients is a key area of physical stability during end-of-life care [4]. The deterioration of the skin structure and reduction of its functions and the associated gradual loss of skin integrity is a natural phenomenon of aging [5, 6]. Furthermore, other disease-associated skin weakening, such as cancerous wounds, as well as wound-like pressure injuries that occur due to immobility, nutritional imbalances, and cracks in the skin, make the elderly key subjects of wound care [7, 8]. The purpose of wound care for patients at the end of life is no longer healing, but rather alleviating symptoms; this is referred to as palliative wound care [2].

Palliative wound care is defined as the integration of strategies relating to the prioritization of symptom relief and improvement of a patient’s quality of life [9]. Palliative wound care refers to the maintenance of skin integrity and the management of symptoms such as odors, exudates, pain, bleeding, and infections.

Such care also improves the psychological stability of end-of-life patients, as well as their physical well-being, and facilitates other aspects of terminal care [2, 9]. Therefore, with a growing demand for, and importance of, end-of-life care in healthcare systems like nursing homes, there is a growing demand for nurses to perform effective palliative wound care, leading to the continued publication of related studies. In the study of Emmons and Lachman (2010), a concept analysis was conducted to make the term of palliative wound care easier to use in clinical and research settings. They defined palliative wound care as a holistic integrated approach to care that addresses symptom management, is interdisciplinary/multidisciplinary, is driven by patient/family goals, and considers the patients’ psychosocial well-being in care to advance their quality of life [10]. Woo (2015) suggested that palliative wound care should start with assessing the whole person and than evolve to balancing the palliative patient’s individual care needs and the patient’s circle of care [11]. Wound-related studies, books, and guidelines also guide palliative wound care, and their scope and volume are increasing [12, 13]. However, since some studies and guidelines convey only theoretical knowledge, it is difficult for nurses to employ them directly in clinical practice [14, 15]. It is necessary to combine theoretical knowledge with wound care nurses’ experiences for use in clinical practice. Because wound care nurses performing palliative wound care are involved in a patient’s end of life, their empirical knowledge derived from experience extends beyond general wound care [9, 11, 16].

To effectively provide palliative wound care for patients at the end of life, it is necessary to build a knowledge structure that confirms the subjective viewpoint gained through experiences in palliative wound care. When approached with a methodology that scientifically studies human subjectivity, the understanding of palliative wound care has the potential to be more comprehensive and concrete [17]. This study thus applies Q-methodology to classify the subjective frames of reference of wound care nurses regarding palliative wound care and to identify the characteristics of each Q-factor type to provide basic data for developing programs that raise nurses’ competencies regarding palliative wound care.

## Methods

This is a descriptive study that precedes an empirical study for palliative wound care by wound care nurses with the goal of utilizing Q-methodology to confirm the subjective frame of palliative wound care. Q-methodology involves the research of in-depth opinions by constructing statements based on various perceptions of a specific subject, aiming to objectively measure subjective human aspects of attitudes, beliefs, and values [17]. The methodology is centered on the subjectivity or types of individual attitudes, which is useful in measuring individual attitudes instead of studying abilities or objective behaviors [18]. As such, it is suitable for this study, which focuses on wound care nurses’ subjective frames on palliative wound care.

### Q-sample selection: constructing the Q-population and determining the Q-sample

The selection of a Q-sample consists of two stages: first constructing the Q-population and then determining the Q-sample [17]. Constructing the Q-population involves the collection of statements through interviews to the point of data saturation, where no further statements could be added [17]. This study collected 118 statements from in-depth interviews on palliative wound care with 10 wound care nurses who had at least 3 years of clinical experience. We excluded statements with overlapping meanings by clarifying any meaning ambiguity, thus selecting a final set of 43 statements that were classified into four attributes derived from the concept analysis of palliative wound care: symptom management, the improvement of psychosocial well-being, a multidisciplinary team approach, and patient/family-driven goals [10]. Last, two researchers with experience in Q-methodology shared their perceptions and finalized a list of 35 statements as the study’s Q-sample to improve the representability and distinctiveness of each category (Table 1).

**Table 1:** Factor arrays for the Q-statements

Q-statements	Factor arrays			
	I	II	III	IV
1. I think it is important to provide education for both patients and relevant people around them since the patients may not be able to do things alone in the future.	1**	4	4	-1**
2. I dress patients’ wounds focusing on how they will look when they die, rather than trying to improve the state of the wounds.	2*	3	−2**	3
3. For managing patient wounds, I prioritize my treatment with strategies from guidelines or research that has been proven to be effective, and I believe these methods are effective.	−4**	0**	2**	4**
4. I believe that it is important to seek feedback by patients on the effectiveness of the strategy rather than stopping at the intervention.	4**	−1	0*	−2
5. I believe that no two patients share the same pathological condition, and that it is important to find and apply methods that fit the patient.	4**	−1**	1	1
6. I choose treatment strategies based on the symptom relief strategies that I have used before with other patients.	3**	−1	0**	−2
7. I do not believe that the recommendations concerning the risk and effectiveness of topical drugs are significant in treating pain from wounds, as they change often.	−4**	0	−1	−3**
8. To alleviate pain, I use thicker dressing products that can reduce pressure rather than drugs that can further deteriorate conditions.	0*	−1	−1	2*
9. I recommend using systemic painkillers whose effects are quick and definitive.	−3	1	1	−3
10. I believe the higher priority is to follow the patient’s wishes to extend or shorten dressing changes to manage exudate or pain.	3**	−2	−2	−1
11. I believe that there are limitations for me in controlling pain through dressings or topical measures and recommend visiting the pain clinic.	−2**	1*	2*	0**
12. I have experienced nutritional problems in patients reaching the terminal stage of their lives and therefore consult with the nutritional department to manage their nutrition.	0	1	3**	−2**
13. I recommend connecting with home caregivers to facilitate consistency in care as patients often need to stay home since it is hard for them to come to the hospital frequently.	0	2*	3**	1
14. I believe that recommending and connecting patients with routes of care in advance are important in ensuring they receive care easily rather than connecting them when their situation has worsened.	0**	4	3	2*
15. I believe that care from non-medical professionals, such as physical therapists and social workers, is more important in palliative wound care at the end of the patient’s life.	0	0	0	0
16. I think there are limitations to what I can do for patients as a wound care nurse since there will be more important things than wound.	−1**	2**	−2	−4
17. I ponder on methods that patients or caregiver can use to deal with dressings, as they may ultimately be done at home or in nursing homes.	1	3	4	1
18. I find it very difficult to listen to patients and guardians asking how they can be cured when the patients cannot be cured.	−1	3**	−1	0*
19. I avoid patients and guardians asking about treatment progress because I do not like talking about negative situations to patients.	−1	2	−1	1
20. I believe that dressings are not an important part of the final journey of the patient and make treatment-focused choices by considering the patient’s financial situation.	2**	−1	0*	−2
21. I choose treatment methodologies as long as the patient’s mind is put at ease by choosing what the patients or their caregivers want.	3	−2*	−3*	0*
22. I believe that the patient must know about their situation accurately to be able to mentally prepare themselves.	−2**	−4	1**	3**
23. When my opinions and the patient’s differ, I invite sufficient dialogue before making a decision rather	2**	0	−1	−1
24. If the patients have the wrong information about a treatment, I believe that they should be presented with the correct information.	−3*	−2*	0**	4**
25. I believe that giving false hope to patients and caregivers is not helpful and let them know that what does not work, does not work.	−2	−3	1**	2**
26. Prior to setting objectives, I believe that the patients and caregivers must be provided with detailed explanations and sufficient time rather than scaring them with negative aspects.	1	0*	1	−1*
27. I prioritize the patients’ opinions over the caregivers’, provided that the patient is conscious, as it is a choice that they make for the last part of their lives.	1**	−3	−3	−4*
28. I cannot feel a sense of achievement with patients receiving palliative wound care	−1**	1**	−4	−3
29. Rather than presenting solutions to terminal stage patients, I believe that it is better for the patients’ stability to listen to their stories	2**	0	0	−1
30. I believe that professional treatment is necessary for psychological stability and recommend referral to a psychiatric clinic.	−2**	0*	2*	1*
31. I believe that the patient should regard the disease and wound process directly and accept it for their own psychological stability.	−3**	−4**	2**	3**
32. I try to avoid saying hopeful things, as they may grow more anxious if they develop hope and then are disappointed.	−1**	1**	−3**	0**
33. I try to tell them things that may provide them with positive strength, such as compliments for their current behavior.	1	−3**	0	0
34. I try to do my best in treating patients so that I do not regret it after they die.	0**	2*	−2**	2*
35. I tell the patients that not being cured is not always unfortunate.	0	−2	−4	0

* *P* < 0.05, ** *P* < 0.01

### P-sample selection

The P-sample is the group of participants who participate in Q-sorting [17]. As the Q-methodology deals with differences between individual internal meanings rather than individual differences, it is not limited by the number of P-samples. Rather than relying on probability-driven sampling methods, P-samples should be conveniently sampled, employing samples that are expected to hold various perceptions of the subject being studied [19]. This study selected a P-sample that includes 40 wound care nurses who agreed to participate in the study, which was conducted between November 2019 and May 2020. The selection criteria included nurses who had worked for more than 1 year as a wound care nurse in a palliative care unit and had completed the Fellowship of Korean Wound Academy program, which includes a palliative wound care section.

### Q sorting

**Fig. 1:**
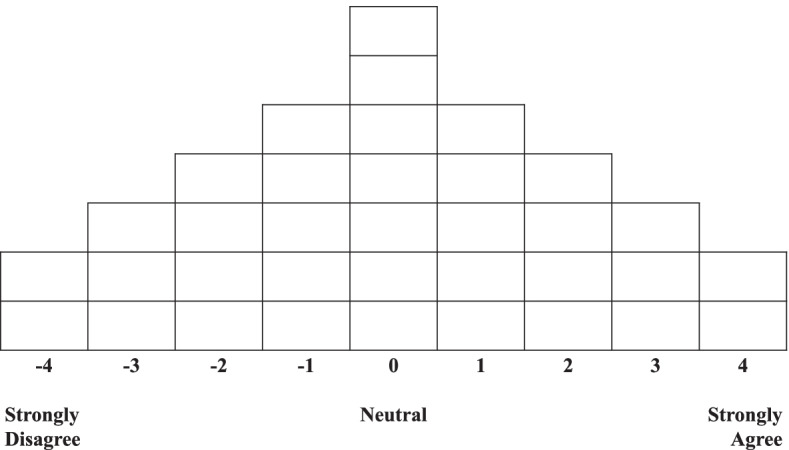
The Q-sorting distribution chart

Q-sorting is a process in which a P-sample classifies Q-samples and assigns points to each item by forced distribution [17]. To achieve this objective, the Q-sample statements were put onto 8 × 3 cm cards for classification. The Q-sorting for this study involved the complete listing of the Q-samples distributed according to a 9-point scale (− 4 to 4) in the form of a quasi-normal distribution, ranging from strong disagreement to strong agreement (Fig. 1). The classification process involved providing the Q cards and the Q-sorting distribution chart to the participants and the participants listening to the researchers’ explanations of the material. Then, the participants placed the cards into three levels: agree, neutral, and disagree. These cards of three levels were sorted into a 9-point scale with the Q-sorting distribution chart. After the classification was completed, we collected data on the demographic characteristics of the participants and their distribution rationale through the P-sample and in-depth interviews conducted by the researchers.

### Data analysis

The coding method for the data collected through the Q classification involved reassigning scores from 1 to 9, assigning 1 point to the two Q cards in the − 4 section, and assigning 9 points to the two cards in the 4 section. These reassigned scores were input into the PQMethod 2.35 program (Schmolck, 2014) to calculate the correlation between P-samples, and 4 Q-factors were derived from the varimax rotation and principal component factor analysis [20, 21].

### Ethical considerations

This study was approved by the institutional review board at the authors’ institution (code: *KUIRB-2019-0274-01).* The purpose and the contents of the study were explained to the participants through a research guide document. The participant consent form stated that they were participating in the study voluntarily. The participants were also told that their responses, including the recordings, would not be used for any purposes other than research and that their confidentiality would be guaranteed, after which they provided written consent. To guarantee the anonymity of the participants, individual information was regarded as confidential, and this private information was protected using identifier codes.

## Results

### Formation of subjective frames on palliative wound care by wound care nurses

This study conducted a factor analysis for the data collected in the study, leading to the formation of four Q-factors. The explanatory power of each Q-factor was 28% for Q-factor I, 18% for Q-factor II, 15% for Q-factor III, and 8% for Q-factor IV, cumulatively explaining 70% of the total variance. Furthermore, the P-samples for each type were 13, 8, 10, and 9 participants, respectively, and the eigenvalues for each type were 11.34, 7.37, 6.09, and 3.23, respectively. Q-methodology is designed to identify types, but not to understand the proportional distribution of each type. In Q-methodology, a Q-factor could sufficiently be explained with 4–5 participants in each Q-factor and that Q-factors with an eigenvalue of 1.0 or higher are statistically significant with explanatory power [17]. As the Q-factors identified in this study each had more than 5 participants and the eigenvalues of each Q-factor were higher than 1.0, the Q-factors were considered significant.

As shown in Table 2, the P-sample characteristics of each Q-factor were generally evenly distributed. The participant with the highest factor loading values for each Q-factor can be considered as the participant who is representative of that Q-factor, demonstrating its most typical characteristics.

**Table 2:** Characteristics for the P-sample

Q-factor		I (*n* = 13)	II (*n* = 8)	III (*n* = 10)	IV (*n* = 9)
Age (years)		31.38 ± 3.07	31.63 ± 1.85	37.40 ± 4.33	37.11 ± 2.26
Gender (n)	Female	13	8	10	9
	Male	0	0	0	0
Education (n)	BSN	7	3	0	0
	MSN	6	5	7	8
	Doctorate	0	0	3	1
RN experience (years)		6.92 ± 3.09	7.25 ± 2.05	13.20 ± 4.21	12.44 ± 2.51
WCN experience (years)		1.15 ± 1.28	3.88 ± 1.13	7.90 ± 2.13	7.78 ± 2.77
PWC experience (years)		1.01 ± 1.11	3.88 ± 1.13	6.77 ± 2.09	6.00 ± 2.18

*Abbreviations*: *BSN* bachelor’s degree in nursing, *MSN* master’s degree in nursing, *RN* Registered nurse, *WCN* wound care nurse, *PWC* palliative wound care

### Analysis of subjective frames on palliative wound care by wound care nurses

The four subjective frames on palliative wound care by wound care nurses are as follows: “Focusing on care within the boundary of current patient demands,” “Comparing continuously the priorities on wound healing and disease care,” “Preparing and preventing from worsening via tracking care in advance,” and “Moving forward with a clear direction by confronting the declining condition.”

### Q-factor I: focusing on care within the boundary of current patient demands

Q-factor I included the P-samples of 13 participants; Q-statements with which this group strongly agreed were 4(+ 4), 5(+ 4), 6(+ 3), and 10(+ 3), and the Q-statements with which this group strongly disagreed were 3(− 4), 7(− 4), 24(− 3), and 31(− 3) (Table 1). This Q-factor type does not include the belief that any two patients are the same; while patients may have the problem of wounds in common, the nurses consider that the Q-factors influencing the wounds all differ and take a therapeutic approach that considers the conditions and feedback of individual patients. Furthermore, as the purpose of palliative wound care is patient well-being, this type places the foremost priorities on patient demands and convenience and on ensuring that the patients are comfortable. At the same time, they do not consider standardized knowledge, such as guidelines and recommendations, as important. This suggests that rather than focusing on such formulas, they concentrate on the treatment of inconveniences felt by the patient and that their approach to palliative wound care is centered on the alleviation of symptoms rather than on the treatment of wounds.

The reasons that participant 25, who had the highest Q-factor weightings in this type, chose the cards of strongest agreement and disagreement are as follows: “When managing pain throughout symptom management, I rearrange schedules if the patient tells me that they cannot do it on that day due to too much pain, even if (new) dressings were scheduled for that day. If there is anything that the patient asks for, telling me, ‘This is what I find comfortable,’ I try to do as the patient says as much as possible, even if it really does not make sense.”

### Q-factor II: comparing continuously the priorities on wound healing and disease care

Q-factor II included the P-samples of 8 participants; the Q-statement with which this group strongly agreed with was 18(+ 3), and the Q-statements with which this group strongly disagreed with were 31(− 4) and 33(− 3) (Table 1). This type considered the systemic conditions of the body to be an important component in wound care and sought methods of wound care depending on the body’s conditions. However, they also demonstrated avoidance of the situation, as they felt guilty that they were unable to actively engage in wound care since patients at the end of their lives faced a declining ability to heal wounds. This type found interactions with patients difficult, whether it was disappointing the patients when directly explaining their circumstances or giving them hope by talking to the patients positively. Furthermore, they tended to engage in wound care in accordance with the patients’ body conditions.

The reasons that participant 8, who had the highest factor weightings among this type, chose the cards of strongest agreement and disagreement are as follows:

“There are times when what we think and what the caregivers and the patients think differ. The patients and caregivers tend to focus on wounds that are visible on the surface when their internals are becoming even more ruined. Even if the pressure injury is treated actively, the patients will not recover if their body conditions do not improve. Curing the pressure injury is not what is important right now. I think that the best method for wound care is to monitor the patient’s body conditions and tailor the treatment methods to the monitoring results.”

### Q-factor III: preparing and preventing from worsening via tracking care in advance

Q-factor III included the P-samples of 10 participants; the Q-statements with which this group strongly agreed with were 12(+ 3) and 13(+ 3), and the Q-statements with which they strongly disagreed with were 21(− 3) and 32(− 3) (Table 1). This type seeks to identify in advance factors or problems that may affect wound care for patients as they reach the end of their lives. They place importance on cooperation and the exchange of opinions, not only between patients and caregivers, but also with various healthcare personnel, such as nutrition teams and home nurses. Therefore, this type tends to have an open attitude towards patients, caregivers, and other healthcare personnel, shares opinions with them, and attempts to resolve issues that may arise in the future.

The reasons that participant 1, who had the highest factor weightings among this type, chose the cards of strongest agreement and disagreement are as follows:

“When it comes to palliative wounds, reevaluations are necessary when pathological situations of patients change—sometimes retrying chemotherapy, changing the types of drugs, and more. Wounds are impacted by these changes in medical conditions, and it is necessary to have multidisciplinary communication about the patient. It is also necessary to consider nutrition and home care that will later become problematic at the patient’s end of life and helping the patients to adapt to them early on. Therefore, I believe our role is to use diverse resources to actively assess, intervene, and connect with the patient.”

### Q-factor IV: moving forward with a clear direction by confronting the declining condition

Q-factor IV included the P-samples of 8 participants; the Q-statements with which this group strongly agreed with were 3(+ 4), 24(+ 4), 22(+ 3), and 31(+ 3), and the Q-statements with which they strongly disagreed with were 27(− 4), and 7(− 3) (Table 1). This type believes in the proper treatment of palliative wounds based on clear guideline for wound care. Therefore, they also believe that patients should be fully aware of their conditions. To ensure clear and efficient judgment regarding wound treatment, wound care nurses believe that they should provide proper guidance since they possess the most knowledge, especially compared to patients or caregivers who may be emotional.

The reasons that participant 27, who had the highest factor weightings among this type, chose the cards of strongest agreement and disagreement are as follows:

“I tell the patients from the beginning rather than lying or evading details about their progress. I think that psychological well-being involves difficulties and resistance until patients can face their condition and accept it, but once patients accept it, patients are able to see it directly and be prepared, so I think we should be firm with the patients. It is very important to accurately describe the current condition of the patient and to give the options of various wound care methods. Wound care nurses should do their best to give wounds a chance to heal with expert knowledge.”

## Discussion

Palliative wound care is a holistic and integrated approach to treatment for alleviating the symptoms of patients with chronic wounds and improving their quality of life, irrespective of whether their wounds can be cured. In this integrated approach, treatment is influenced significantly by the perceptions and subjective frames of reference of wound care nurses towards palliative wound care.

From the results of this study, a common trait shared by all four Q-factors is the perception of palliative wound care as an approach to improving the quality of life of end-of-life patients that includes holistic patient care, family support, effective communication, and interdisciplinary teamwork. This is reflected in the priority placed on palliative care as a crucial part of integrated, people-centered health services [22].

In particular, the findings of this study illustrate that decisions based on assessments about what should take priority in wound and disease processes is an important axis in palliative wound care. This is reflected in Q-factors II and IV. Q-factor II emphasized the constant reconfiguration of treatment objectives so that the body’s condition is not compromised rather than specifically focusing on wound treatment. Given that the majority of patients are end-of-life patients in need of palliative wound care, their wounds are often only treated to stop them from worsening [23]. To effectively administer wound care to end-of-life patients, nurses must assess and consider such treatment in a holistic manner that include the progression of a patient’s disease(s) as they approach death [9]. Therefore, since a nurse who provides palliative wound care must also possess an overall knowledge of the patient’s disease, education on this must be done together with wound treatment training. On the other hand, Q-factor IV emphasized wound care and the expert role of wound care nurses. This reflected that proven, professional opinions lead to effective treatment processes, rather than decision-making processes involving patients or their caretakers [24, 25]. This view emphasizes the vital role of the problem solver, with wound care nurses providing professional leadership. As experts and leaders, they must be able to use their experiences to provide clear evidence for proper research [26]. Furthermore, it is necessary to develop a palliative wound care protocol by compiling evidence so that all nurses are able to effectively engage in wound care. While this protocol may be similar to what is used in general wound care, the objectives and processes must differ in palliative wound care [27]. This should result in balancing the burdens of and benefits to patients, resulting in treatment objectives merging with the goal of raising patients’ quality of life. Even if the protocol is guided by the precepts of beneficence and the best scientific evidence, what may be a priority for the nurse may not be a priority for the patient and family. We should consider that what is considered right or wrong for end-of-life patient care is not always important.

In addition to the disease and wound status of patients, the findings of this study also illustrate that timeline is an important axis in palliative wound care. This is reflected in Q-factors I and III. Q-Factor I emphasized respecting the present demands of patients. Nurses in this Q-factor perceived that patient-centric healthcare is possible when decision-making is driven by the values of the patient. These Q-factor perceptions are reflected in previous studies, which found that patients are invited to participate in the selection and determination of treatment, especially in the case of palliative care [28, 29]. It seems necessary to develop a tool to aid patient self-reporting and invite active participation by the patients, allowing them to communicate their symptoms and demands to the medical staff. Furthermore, it would be important to provide education and facilitate an environment that encourages empathetic tendencies and listening carefully and sympathetically to patients. Q-factor III emphasized forecasting patient progress based on past and present experiences and believe that a variety of healthcare professionals should work to ensure patients’ future comfort, while Q-factor I mainly focused on their present condition. Q-factor III prefers a supportive environment and an integrated approach to treatment through sharing common objectives, sharing information, and providing mutual support for preparing needed care in the future. The need for a multidisciplinary approach to palliative wound care has been emphasized in many existing studies. Such approaches have been found to increase the satisfaction of medical personnel since they encourage communication within the larger framework of patient-centered care [30]. Possible disarticulation between professionals involved in comprehensive care and possible disarticulation between the levels of care, mainly hospital and home care, could be reasons for the problem of transferring knowledge to practice. To advance multidisciplinary cooperation in palliative wound care, it is necessary to hold conferences or provide education to ensure fluid communication and unified thinking between disciplines and to develop tools and systems for efficient communication.

The limitations of this study are that because the participants were recruited using convenience and localized sampling, the results are difficult to generalize. However, the Q-methodology as a research method aims to understand the meaning of a phenomenon for the participants who experience it rather than to generalize the research findings.

**Fig. 2:**
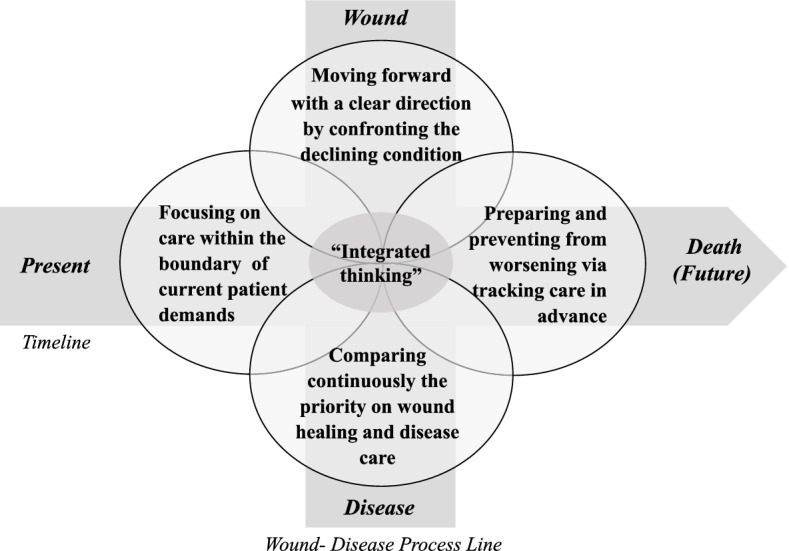
Subjective frame of palliative wound care by wound care nurses

This study identified how wound care nurses structure the subjective frames on palliative wound care by using Q-methodology. As this method focuses on individual subjectivity, this study does not intend to classify the types of subjective frame identified in this study as either positive or negative. Rather, it is necessary to conduct care that considers the patient’s present to future and that integrates wound state and disease progression for implementing effective palliative wound care across all types (Fig. 2). By identifying the types of subjective frames on palliative wound care among wound care nurses, this study contributes to the knowledge related to practical nursing. We hope that it will provide a foundation for education and future research, raising the competencies of palliative wound care in nurses and improving the quality of treatment and life in patients receiving palliative care.

## Conclusion

Understanding the subjective viewpoints on palliative wound care constructed by expert practice is important for successful wound care at the end of life. This study confirmed 4 types of subjective frames on palliative wound care among wound care nurses. The results of this study can be useful in developing specific nursing strategies for palliative wound care by all nurses involved in palliative care, not only wound care nurses. We expect that the subjective viewpoints identified and discussed in this study, as well as type-specific reinforcement measures, can be the basis for developing practical palliative nursing materials and effective tools in clinical settings.

## Data Availability

The datasets generated and/or analyzed during the current study are not publicly available due to [individual privacy could be compromised] but are available from the corresponding author on reasonable request.
